# Patent abdominal subcutaneous veins caused by congenital absence of the inferior vena cava: a case report

**DOI:** 10.1186/1752-1947-4-223

**Published:** 2010-07-23

**Authors:** Wolfgang J Schnedl, Pia Reittner, Robert Krause, Rainer W Lipp, Erwin Tafeit, Sandra J Wallner-Liebmann

**Affiliations:** 1 Department of Internal Medicine, Medical University of Graz, Auenbruggerplatz 15, A-8036 Graz, Austria; 2 Practice for General Internal Medicine, Hauptstrasse 5, A-8940 Liezen, Austria; 3 Diagnostikum Sued-West, Weblinger Guertel 25, A-8054 Graz, Austria; 4 Institute of Physiological Chemistry, Centre of Physiological Medicine, Harrachgasse 21/II, A-8010 Graz, Austria; 5 Institute of Pathophysiology, Centre for Molecular Medicine, Heinrichstrasse 31a, A-8010 Graz, Austria

## Abstract

**Introduction:**

Patent paraumbilical and abdominal subcutaneous veins are found frequently as collaterals in patients due to portal hypertension mainly in liver cirrhosis.

**Case presentation:**

For evaluation of portal hypertension in a 72-year-old Caucasian man without liver cirrhosis, magnetic resonance imaging with gadolinium contrast-enhancement was performed and demonstrated a missing inferior vena cava. A blood return from the lower extremities was shown through enlarged collateral veins of the abdominal wall, vena azygos and hemiazygos continuation, and multiple liver veins emptying into the right cardiac atrium. We describe a rare case of abdominal subcutaneous wall veins as collaterals caused by a congenitally absent infrarenal inferior vena cava with preservation of a hypoplastic suprarenal segment.

**Conclusion:**

Knowledge of these congenital variations can be of clinical importance and it is imperative for the reporting radiologist to identify these anomalies as they can have a significant impact on the clinical management of the patient.

## Introduction

Congenital malformations of the inferior vena cava (IVC) are rare. Patients are usually asymptomatic and this developmental anomaly is detected incidentally during abdominal surgery or radiologic evaluation. Patent paraumbilical and abdominal subcutaneous veins are found frequently as collaterals in patients due to portal hypertension, mainly associated with liver cirrhosis [1,2]. In this particular patient, abdominal sonography and liver function laboratory parameters revealed no signs of liver cirrhosis. For further evaluation of portal hypertension, magnetic resonance imaging of his abdomen was performed and demonstrated absence of the inferior vena cava. Thus, we describe patent abdominal subcutaneous wall veins as collaterals in a patient with congenital absent IVC with preservation of a hypoplastic suprarenal segment.

### Case presentation

A 72-year-old Caucasian man reported no symptoms at a preoperative evaluation before hip joint replacement. Upon his admission, abnormal laboratory parameters included erythrocyte sedimentation rate (ESR) 29/73mm/hour (normal < 10) and total cholesterol 233 mg/dL (normal < 200); all other routine laboratory parameters including those for liver function, were within normal limits. During clinical examination, a distended patent abdominal wall and paraumbilical veins were noted. An additional abdominal sonography revealed no signs of liver cirrhosis. During anamnesis, he reported a history of deep vein thrombosis in both lower limbs at age 50, and recurring thrombosis at age 62 of unknown etiology.

**Figure 1:**
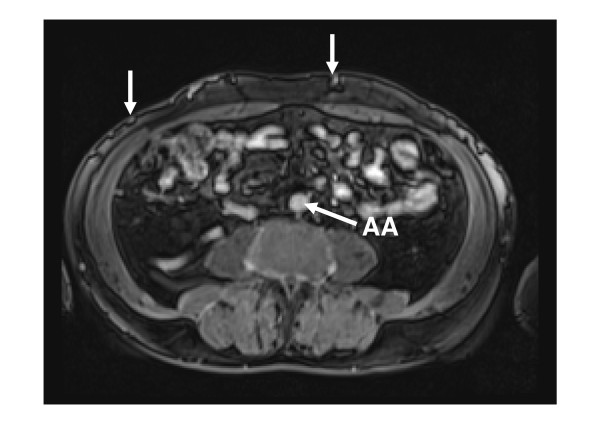
**Gadolinium-enhanced magnetic resonance imaging (MRI) of the abdomen at infrarenal level showing abdominal wall veins and absence of the inferior vena cava**. AA, abdominal aorta

**Figure 2:**
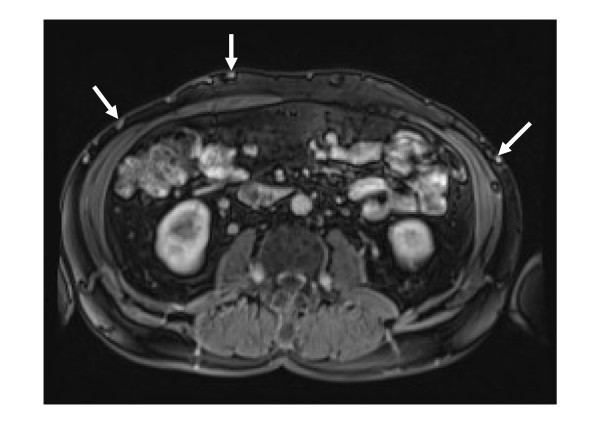
**Gadolinium-enhanced MRI showing multiple paraumbilical and abdominal wall veins**.

**Figure 3:**
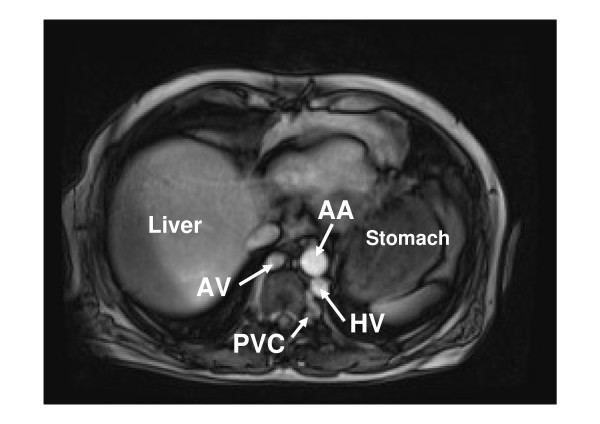
**MRI demonstrating the hemiazygos vein (HV) and the azygos vein (AV) enlarged to compensate this anomaly**. They collect the blood return from dilated paravertebral venous collaterals (PVC).

**Figure 4:**
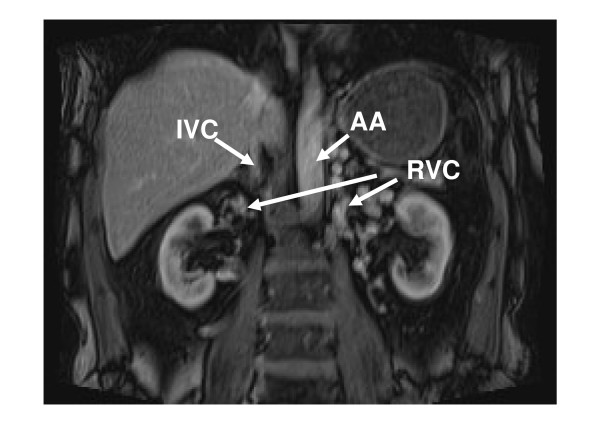
**MRI showing renal vein collaterals (RVC) collecting venous blood return from both kidneys**. AA, abdominal aorta; IVC, inferior vena cava

**Figure 5:**
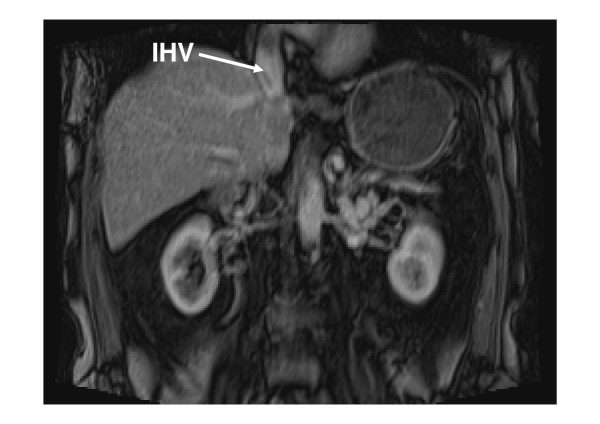
**Coronal MRI showing the intrahepatic veins (IHV) empty directly into the right cardiac atrium**.

Magnetic resonance imaging (MRI) of the abdomen with gadolinium contrast enhancement was performed and the infrarenal IVC could not be demonstrated (Figure 1). The MRI showed multiple patent abdominal wall veins (Figure 2) and enlarged ascending lumbar veins were shown as collaterals. Blood return from the lower extremities was seen through enlarged vena azygos and hemiazygos (Figure 3). The intrahepatic IVC was hypoplastic with a maximum size of 1 × 0.4 cm (Figure 4). Liver veins were seen to empty directly into the right cardiac atrium (Figure 5). No concomitant visceral or cardiac pathologic findings were noted. Screening for thrombophilia, including activated protein C resistance time, factor V mutation Leiden, levels of protein C and S, presence of antithrombin III, and phospholipid antibodies revealed no reason for thrombosis.

The hip replacement was performed successfully using anticoagulation with low-molecular weight heparin; he was subsequently anticoagulated with coumarin and compression stockings were prescribed. Although familial clustering of congenital anomalies of the IVC is known, his family members refused further investigations. Written informed consent was obtained for all procedures, which were in accordance with the Declaration of Helsinki and the recommendations of the local ethics committee.

## Discussion

The umbilical vein is obliterated a few days after birth and is usually not part of the actual circulation. Patent paraumbilical and abdominal subcutaneous veins are found frequently as collaterals due to portal hypertension in liver cirrhosis or with abdominal and retroperitoneal tumors [3]. Congenital and acquired abnormalities of the inferior vena cava are known [4], but only a few more than 50 case reports and small case series of deep vein thrombosis (DVT) and anomalous IVC are reported in the current English-language medical literature [5]. It is likely that the incidence of IVC is underestimated, and limited case series have reported a high estimated prevalence of up to 8% in the general population [6]. In infants these development anomalies may be combined with heart and visceral malformations [7]. Without other associated congenital defects, the absence of an IVC can be asymptomatic. The discovery is usually incidental during abdominal imaging procedures or abdominal surgery.

The IVC develops between the sixth and eighth week of embryonic life as a composite structure formed from the appearance, regression, and anastomosis of three paired embryonic veins: the posterior cardinal, subcardinal, and supracardinal veins. A normal IVC is composed of four segments which are hepatic, suprarenal, renal and infrarenal. Variations of IVC anatomy are classified in a system based on abnormal regression and/or persistence of various embryonic veins [8]. We describe a congenitally absent infrarenal IVC with preservation of a hypoplastic suprarenal segment causing abdominal subcutaneous veins as collaterals. With this anomaly, the external and internal iliac veins join to form enlarged ascending abdominal wall and lumbar veins, which convey blood return from the lower extremities to the azygos and hemiazygos veins via anterior paravertebral lumbar veins. The absence of the entire posthepatic IVC is demonstrated through multiple hepatic veins which empty into the right atrium (Figure 5). This suggests the very rare case that all three paired venous systems may have failed to develop properly. However, since it is difficult to identify single embryonic events, it is somewhat unclear whether these conditions are true developmental anomalies or the result of pre-or perinatal thrombosis [8].

The course and number of collateral veins are variable and, due to inadequate collateral circulation, this results in venous stasis and an increased risk of DVT. Congenital anomalies of the inferior vena cava are described as a rare cause of thrombotic occlusions in iliac veins usually in patients 30 years of age and younger. In medical outpatients, the mean age of DVT (± SD) was reported as 59.1 (± 17.3) years, which compares well to the age DVT occurred and routine management for DVT was performed in this patient. As commonly recommended for DVT, oral anticoagulation in this patient was discontinued three to six months after both the first and second occurrence of thrombosis. However, after thrombosis is caused by absence of IVC, there is a reportedly high risk of recurrent thrombosis [9]. The combined occurrence of IVC malformation and presence of heterozygous factor V Leiden mutation may represent an even higher risk for DVT. Although there is no standard therapy established, it is suggested that patients with congenital anomalies of the inferior vena cava should be anticoagulated for their entire lifetime [6,7].

## Conclusion

Nowadays, vascular anomalies can well be identified with venography, improved contrast-enhanced computed tomography, and high resolution magnetic resonance imaging methods [5,8,10,11]. MRI helps to demonstrate the course of collateral pathways and distinguish aberrant vessels from tissue masses. Although congenital malformations of IVC are a rare cause of thrombosis, the right diagnosis may help to avoid unnecessary surgery, and lifetime anticoagulation avoids recurring thrombosis [6,12]. However, knowledge of these congenital variations can be of clinical importance and is essential to avoid diagnostic pitfalls. Therefore, it is imperative for the reporting radiologist to identify these anomalies, as it can have a significant impact on the clinical management of the patient.

## Consent

Written and informed consent was obtained from the patient for publication of this case report and accompanying images. A copy of the written consent is available for review by the Editor-in-Chief of this journal.

## Competing interests

The authors declare that they have no competing interests.

## Authors' contributions

WJS and RWL conceived of and designed the study. WJS and PR analyzed and interpreted the data. WJS drafted the article and, along with ET and SJW, critically revised it for important intellectual content. WJS, ET, SJW, RK and RWL approved the final article. WJS, RK and RWL provided administrative, technical, or logistic support. WJS collected and assembled the data. All authors have read and approved the final manuscript.
